# Gut microbiota and ankylosing spondylitis: mechanisms, functional pathways, and research trends

**DOI:** 10.3389/fmicb.2026.1828220

**Published:** 2026-05-13

**Authors:** Zhe Liu, Ningning Li, Hao Zhang, Donglin Hao

**Affiliations:** 1 Department of Rheumatology and Immunology, Zhumadian Central Hospital, Zhumadian, Henan, China; 2 Department of Rheumatology and Immunology, Central Hospital of Dalian University of Technology, Dalian, China; 3 Department of Rheumatology and Immunology, Suzhou TCM Hospital Affiliated to Nanjing University of Chinese Medicine, Suzhou, China

**Keywords:** ankylosing spondylitis, gut microbiota, gut–joint axis, host–microbe interactions, Mendelian randomization

## Abstract

**Background:**

Ankylosing spondylitis (AS) is a chronic immune-mediated inflammatory disease in which genetic susceptibility, mucosal immunity, and environmental factors converge. Growing evidence indicates that gut microbiota dysbiosis is closely involved in AS pathogenesis, yet the evolution of this research field and the underlying functional mechanisms remain to be systematically clarified.

**Methods:**

The study performed an analysis of studies on AS and gut microbiota retrieved from the WOSCC, Scopus, and PubMed. Publication trends, collaboration networks, co-citation patterns, and keyword clusters were analyzed to identify major research themes and emerging hotspots in this field.

**Results:**

The analysis revealed a progressive shift from descriptive microbiota profiling to mechanistic and causal investigations. Core research themes included microbial dysbiosis, intestinal barrier dysfunction, mucosal immune activation, microbial metabolites, and key inflammatory pathways. Studies increasingly emphasize functional and pathway-level analysis rather than focusing on individual microbial taxa. Mendelian randomization further strengthened causal inference and highlighted the potential of microbiota-related signatures for disease stratification and therapeutic response.

**Conclusion:**

These findings support a disturbed gut–joint axis as a central feature of AS and underscore the role of functional microbial pathways in immune dysregulation. Integrating standardized multi-omics data with causal validation and refined clinical phenotyping may facilitate the identification of actionable microbial targets and advance microbiota-informed precision strategies for AS.

## 1 Introduction

Ankylosing spondylitis is a chronic immune-mediated disorder characterized by axial joint inflammation, enthesitis, and progressive structural damage (Sieper and Poddubnyy, 2017). Despite the well-established genetic link between HLA-B27 and AS (Zhang et al., 2024), genetic factors account for less than one-third of disease susceptibility, indicating that other determinants are critically involved in AS pathogenesis and progression. Driven by advances in high-throughput sequencing, metagenomics, and mucosal immunology, AS research has shifted from a focus on genetics and local joint inflammation to a more comprehensive framework of the microbiota-gut-immune-joint axis. Microbial dysbiosis, impaired intestinal barrier function, and abnormal mucosal immune activation collectively contribute to disease progression and have emerged as key research priorities in recent years.

Increasing evidence indicates that patients with AS commonly exhibit gut microbial dysbiosis. Patients with AS show decreased abundance of short-chain fatty acids (SCFAs)-producing bacteria, including *Faecalibacterium prausnitzii*, Roseburia, and Blautia, which generally exert anti-inflammatory effects and support mucosal homeostasis. Conversely, pro-inflammatory bacteria, including Prevotella, Proteobacteria, Megamonas, *Ruminococcus gnavus*, and taxa within Lachnospiraceae and Ruminococcaceae, are more abundant. Notably, Dialister, an inflammation-related genus, is positively correlated with disease activity, suggesting its potential utility as an inflammatory biomarker or therapeutic target (Ng and Lassere, 2025; Yin et al., 2020; Min et al., 2023; Berland et al., 2023; Breban et al., 2017). The extent of microbial depletion correlates positively with disease activity, indicating that the structural integrity of the gut microbiota may be critically involved in modulating inflammatory progression (Mauro et al., 2025; Lobiuc et al., 2025; Yemula and Sheikh, 2024; Su et al., 2024). Notably, the overall pattern of these microbial changes closely resembles that seen in inflammatory bowel disease (IBD) and psoriatic arthritis, implying that AS may share underlying mucosal immune abnormalities with other immune-mediated disorders.

The intestinal epithelial barrier, a critical interface between gut microorganisms and the host immune system, plays a central role in the pathogenesis of AS. Evidence indicates that AS patients exhibit reduced tight junction (TJ) protein expression and increased zonulin levels, resulting in epithelial barrier dysfunction that allows microbial components, including lipopolysaccharide (LPS), peptidoglycan, and bacterial-derived peptides, to penetrate the lamina propria (Ciccia et al., 2017). Subsequently, recognition of these microbe-associated molecular patterns by dendritic cells and macrophages triggers robust IL-23 production, driving the expansion of Th17 cells, γδT cells, mucosal-associated invariant T cells, and group 3 innate lymphoid cells, which in turn secrete IL-17, IL-22, GM-CSF, and tumor necrosis factor (TNF), thereby establishing the central immunoinflammatory axis of AS (Qaiyum et al., 2021; Gracey et al., 2020; Wei et al., 2025).

In recent years, the number of studies on AS and the gut microbiota has increased rapidly, yet the field still faces several challenges. First, pronounced heterogeneity in study cohorts, sequencing platforms, and analytical methods limits the comparability and reproducibility of findings. Second, the field is inherently interdisciplinary, spanning immunology, microbiology, bioinformatics, and rheumatology, which complicates systematic integration and comprehensive evaluation. Moreover, few studies systematically examine the developmental trends, research hotspots, and thematic clusters of AS-gut microbiota research at the macro level. In this context, bibliometric approaches enable the identification of research hotspots and emerging frontiers by quantitatively analyzing publication trends, international and institutional collaboration networks, author co-citation structures, and keyword co-occurrence patterns. Accordingly, we conducted a bibliometric analysis to systematically assess the body of research in the interdisciplinary field of AS-gut microbiota, delineate the current research landscape, identify key research directions, and inform future mechanistic investigations and microbiota-based intervention strategies.

## 2 Methods

### 2.1 Data collection and search strategy

This study conducted a retrospective bibliometric analysis of publications on the association between AS and the gut microbiota from 1976 to 2026. To obtain comprehensive and high-quality literature data, a systematic search was conducted across WOSCC, Scopus and PubMed. Based on the selected databases, independent yet structurally consistent search strategies were developed. The search focused on two core concepts, namely “Ankylosing Spondylitis” and “Gut microbiota,” which were combined using Boolean operators and wildcards to capture relevant term variations. To enhance topic relevance, the search scope was restricted to title, abstract, and keywords. Specifically, the Topic (TS) field was applied in WOSCC, which encompasses titles, abstracts, and keywords; the TITLE-ABS-KEY field was used in Scopus; and the Title/Abstract field was applied in PubMed. All searches were limited to English publications. Only English-language publications were included to ensure consistency in data extraction and comparability across databases. The final search was conducted on February 18, 2026. The complete search strategies are provided in Supplementary Table 1.

The initial search yielded a total of 531 records (WOSCC: 394 records; Scopus: 128 records; PubMed: 9 records). The type of literature is limited to “Article” and “Review.” Exclude other types of literature such as letters, meeting summaries and other non-research publications. Two independent reviewers independently screened all retrieved records for relevance, with disagreements resolved by a third reviewer. After excluding irrelevant studies, bibliographic data were downloaded in plain text and CSV formats, including full records and cited references. Python (version 3.11) was used to convert Scopus CSV files and PubMed TXT files into a unified plain text format consistent with the WOSCC full record and cited reference structure. Data cleaning was also conducted using Python (version 3.11), including DOI-based deduplication; removal of retracted publications; exclusion of records with “[Anonymous]” in the author field; elimination of virtual institutions such as the Egyptian Knowledge Bank; and standardization and merging of duplicated institution names. Ultimately, 332 publications were retained for subsequent bibliometric analysis.

### 2.2 Data analysis and visualization

Data on publication year, journal titles, countries, institutions, authors, keywords, and references were collected. Bibliometrix was used to characterize citation and reference distributions across journals, and core journals were identified using Bradford’s law. International collaboration networks were then visualized and mapped. Finally, the top 10 most cited publications and references were analyzed. CiteSpace was employed to generate collaboration networks among countries, institutions, and authors, and to conduct reference and keyword burst analyses. In the network maps, nodes indicate publication volume, while centrality reflects relative importance. Keyword co-occurrence analysis was conducted in VOSviewer. Keyword patterns were identified, synonymous terms were merged, and results were visualized using both network and density-mapping approaches.

## 3 Results

### 3.1 Literature and publications

**Figure 1:**
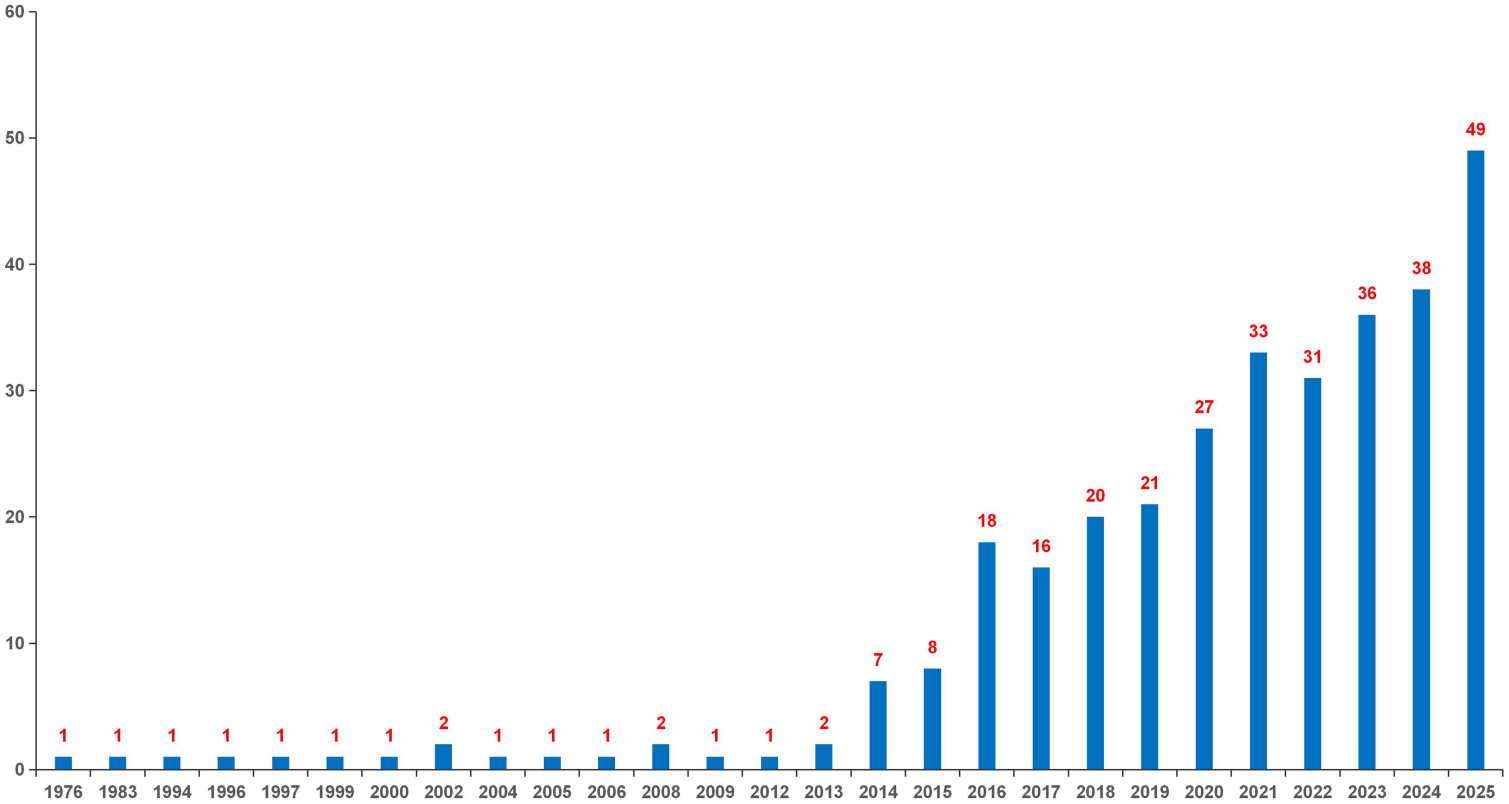
The number of annual publications for AS and the gut microbiota from 1976 to 2025.

Between 1976 and 2026, 332 publications on AS and the gut microbiota were identified. Publications from 2026 were not included in the annual trend analysis due to incomplete data coverage (Figure 1). The exclusion of partial data from 2026 ensured the comparability of annual publication trends. Research in this field has evolved from a slow initial phase to rapid growth: from 1976 to 2013, publication output and citation frequency remained relatively low. Since 2014, research activity has increased substantially, with 49 publications in 2025. From a disciplinary perspective, late 20th-century AS research primarily focused on HLA-B27-associated genetic susceptibility, immunoinflammatory mechanisms, and signaling pathways, while the gut microbiota, as a potential pathogenic or modulatory factor, was not systematically investigated. In the 21st century, advances in high-throughput sequencing, integrative multi-omics analyses, and artificial intelligence have highlighted the role of the gut microbiota in AS pathogenesis, driving rapid growth in this research field.

**Figure 2:**
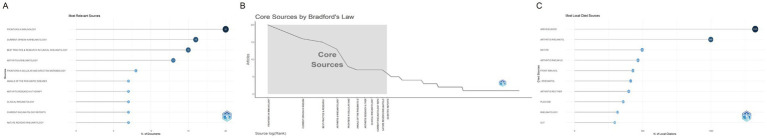
Bibliometric analysis of sources in the field of AS and the gut microbiota. **(A)** The top 10 most relevant sources. **(B)** Core sources by Bradford’s law. **(C)** The top 10 most local cited sources.

The journal distribution analysis showed that Frontiers in Immunology were tied for the most publications, with 20 articles each (Figure 2A). Bradford’s law analysis (Figure 2B) identified 11 core journals (Table 1) that published 113 articles, representing 34.04% of all publications. Notably, Nature Reviews Rheumatology (impact factor 32.7) had the highest impact factor among the core journals, and 81.82% (9/11) of these journals were classified in the JCR Q1 category. The cited journal analysis (Figure 2C) indicated that Annals of the Rheumatic Diseases, Arthritis & Rheumatology, and Nature were the top three journals by citation frequency.

**Table 1:** Top 11 journals of the most publications related to the gut microbiota in the AS.

Rank	Journal	Count	Percentage (%)	Cumulative percentage (%)	IF	Quartile in Category
1	Frontiers in Immunology	20	6.02	6.02	5.9	Q1
2	Current Opinion in Rheumatology	16	4.82	10.84	4.3	Q1
3	Best Practice & Research in Clinical Rheumatology	15	4.52	15.36	4.8	Q1
4	Arthritis & Rheumatology	13	3.92	19.28	10.9	Q1
5	Frontiers in Cellular and Infection Microbiology	8	2.41	21.69	4.8	Q1
6	Annals of the Rheumatic Diseases	7	2.11	23.80	20.6	Q1
7	Arthritis Research & Therapy	7	2.11	25.91	4.6	Q1
8	Clinical Rheumatology	7	2.11	28.02	2.8	Q2
9	Current Rheumatology Reports	7	2.11	30.13	3.9	Q2
10	Nature Reviews Rheumatology	7	2.11	32.24	32.7	Q1
11	Scientific Reports	6	1.81	34.05	3.9	Q1

### 3.2 Countries and institutions

**Figure 3:**
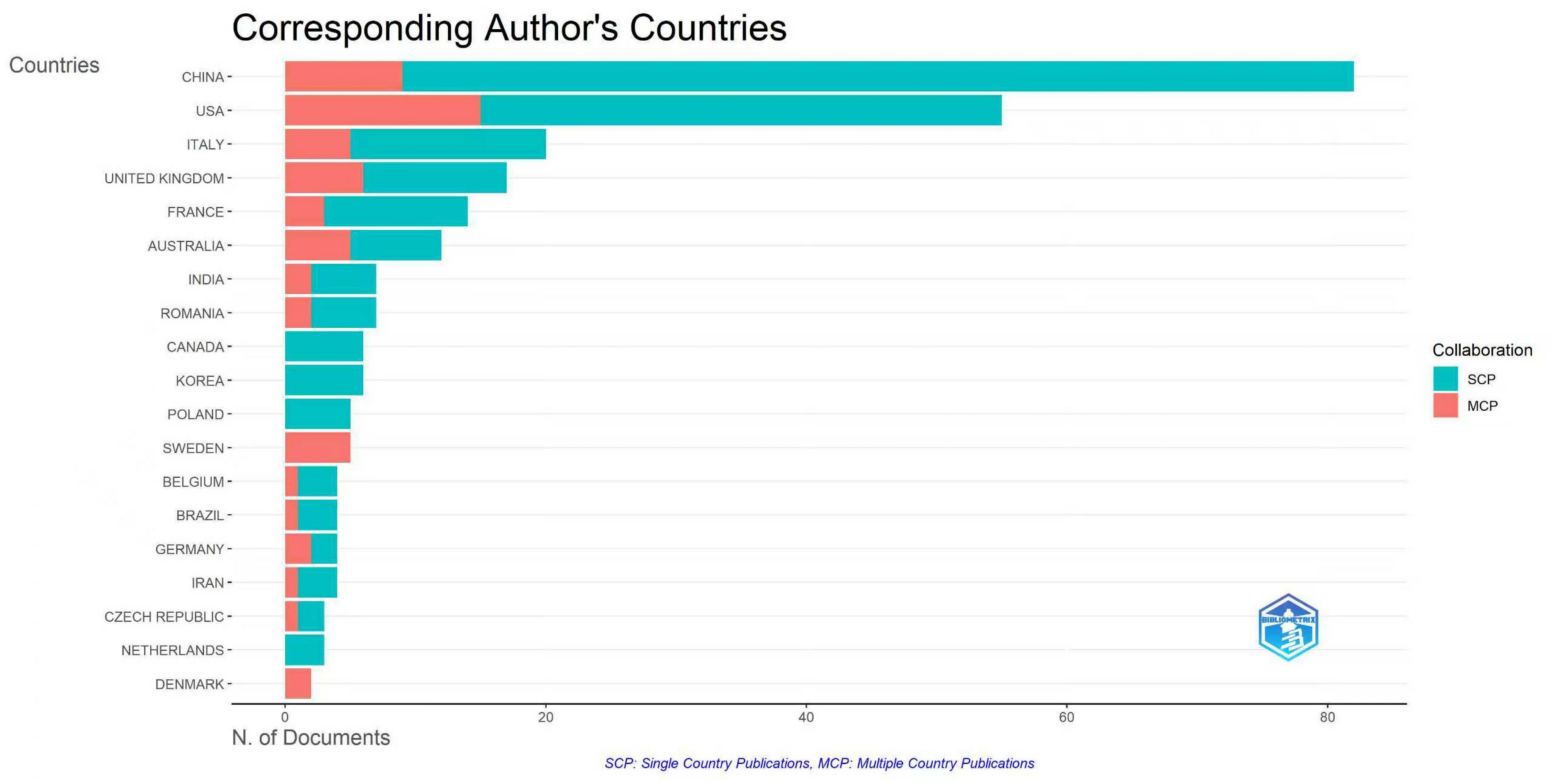
Bibliometric analysis of top prolific corresponding authors’ countries in the field of AS and the gut microbiota. The number of publications is shown for each country, with SCP indicating single-country publications and MCP indicating multiple-country collaborations.

The international collaboration analysis (Figure 3) revealed that Australia (MCP ratio = 0.41), the United Kingdom (0.35), and the United States (0.27) had relatively high proportions of multi-country publications, suggesting that these countries are more inclined to leverage cross-national collaboration to consolidate resources and improve research quality in this field.

**Figure 4:**
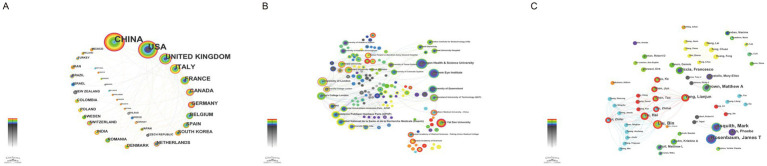
Cooperation network among **(A)** countries, **(B)** institutions, and **(C)** authors. Node size reflects publication output, and links indicate collaborative relationships between nodes. Colored rings indicate the temporal evolution of publications, with outer warm colors representing more recent activity and inner cool colors indicating earlier contributions.

The national collaboration network analysis (Figure 4A) comprised 39 countries and 122 collaborative links. The USA and China contribute the majority of publications. The United Kingdom (centrality = 0.36), USA (0.30) exhibited high centrality, indicating that they serve as key hubs in the global collaboration network, linking multiple research groups and facilitating international academic exchange.

Institutional analysis (Table 2) showed that among institutions with more than seven publications, three were from China. The USA, France, Australia, and the United Kingdom each contributed two institutions. Among these, Oregon Health & Science University ranked first with 20 publications. The teams led by Tejpal Gill and James T. Rosenbaum have long focused on AS research, with particular emphasis on the interaction between HLA-B27 and the gut microbiome, and have exerted substantial academic influence in this field.

**Table 2:** Top 11 institutions according to the total number of publications.

Rank	Institutions	Country	Count	Centrality
1	Oregon Health & Science University	USA	20	0
2	Devers Eye Institute	USA	19	0.02
3	University of Queensland	Australia	13	0.11
4	Institut National de la Sante et de la Recherche Medicale (Inserm)	France	12	0.03
5	Assistance Publique Hopitaux Paris (APHP)	France	11	0.02
6	University of London	UK	9	0.21
7	Sun Yat Sen University	China	9	0.01
8	King’s College London	UK	8	0.13
9	Queensland University of Technology (QUT)	Australia	8	0.11
10	Chinese Academy of Sciences	China	7	0.08
11	Chinese Academy of Medical Sciences-Peking Union Medical College	China	7	0.05

The institutional collaboration network (Figure 4B) comprised 298 institutions and 582 collaborative links, with a relatively loose structure (density = 0.013) and multiple small, relatively independent clusters. As a key node within the University of London (Centrality = 0.21), King’s College London has played a pivotal role in AS and gut microbiota research, cementing its status as a global epicenter for this field.

### 3.3 Authors

The author collaboration network (Figure 4C) showed a dispersed structure, with relatively limited cross-team and international collaboration. The team led by Zhifei Cui, Bin Liu, and Weicong Zhang was represented by blue nodes, reflecting its foundational role in the field’s early development. Meanwhile, the team led by Lianjun Yang, Tao Chen, and Jun Shen was represented by red nodes, indicating high productivity and collaboration in the current stage. Furthermore, within the research group, including Matthew A. Brown, James T. Rosenbaum, and Mark Asquith, James T. Rosenbaum was the most prolific author in this field, having published 19 articles.

### 3.4 Research directions and hotspots

**Figure 5:**
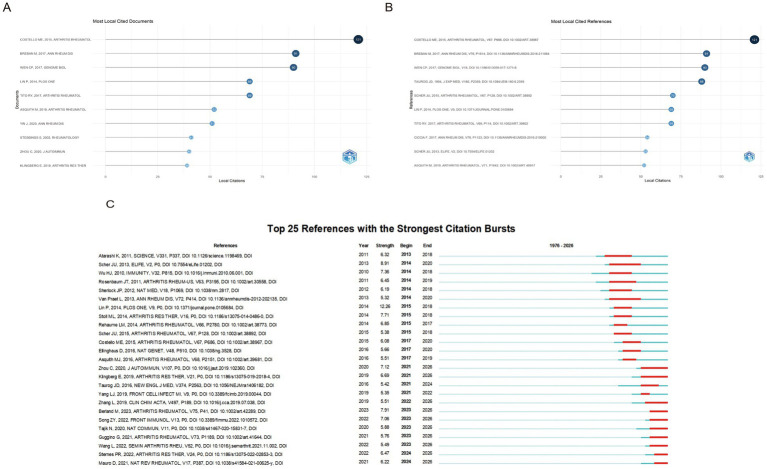
Bibliometric analysis of documents and references in the field of AS and the gut microbiota. **(A)** Most locally cited documents, reflecting the most influential studies within the dataset. **(B)** Most locally cited references, indicating key foundational works frequently cited by the included publications. **(C)** Top 25 references with the strongest citation bursts, highlighting references that have attracted increased attention over specific time periods and represent emerging or rapidly developing research topics. Strength indicates the citation burst intensity, and begin/end denote the start and end years of the burst period. Light blue lines represent the publication time span, while red segments indicate periods of increased citation activity.

The most-cited study by Costello et al. (2015), (Arthritis & Rheumatology) reported distinct differences in terminal ileal microbiota between patients with AS and healthy controls (Figure 5A). Figures 5B,C highlight key references on the association between AS and the gut microbiota. Lin et al. (2014); (Strength = 12.26) showed the strongest citation burst in this field and was a pioneering study demonstrating a direct link between gut dysbiosis and AS in a large-scale clinical cohort. Wu et al. (2010); (Strength = 7.36) investigated the basic mechanisms underlying interactions between the gut microbiota and host immune regulation. Zhou et al. (2016); (Strength = 7.12) showed the strongest recent citation burst and demonstrated alterations in both the composition and function of the gut microbiota in untreated AS patients, uncovering several AS-specific enriched taxa and related metabolic pathways. Klingberg et al. (2019) demonstrated that patients with AS harbor a distinct gut microbiota, and that this dysbiosis was significantly associated with elevated levels of fecal calprotectin. Berland et al. (2023) found that gut microbiota dysbiosis in patients with spondyloarthritis (SpA) was significantly correlated with both disease activity and HLA-B27 status.

### 3.5 Keywords

**Figure 6:**
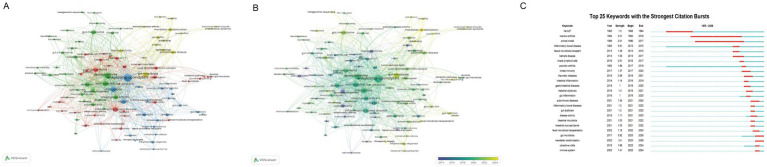
Bibliometric analysis of the keywords in the field of AS and the gut microbiota. **(A)** Co-occurrence network of keywords, showing major research themes and their relationships. Node size represents keyword frequency, links indicate co-occurrence relationships (with thicker lines indicating stronger associations), and different colors represent distinct thematic clusters. **(B)** Timeline distribution of keywords, where colors represent the average publication year, indicating the temporal evolution of research topics. **(C)** Top 25 keywords with the strongest citation bursts, highlighting keywords that have received increased attention over specific periods and reflect emerging research trends.

Keyword co-occurrence analysis (Figure 6A) identified four major thematic clusters in AS-gut microbiota research. The red cluster highlighted immunoinflammatory mechanisms in AS and related immune-mediated disorders, comprising 34 keywords, including inflammatory bowel disease, psoriatic arthritis, and inflammation, indicating a marked overlap between AS and other immune diseases at the level of inflammatory pathways. The green cluster, centered on the relationship between gut dysbiosis and immune-mediated diseases, comprises 33 keywords, including spondyloarthritis, microbiome, and dysbiosis, underscoring the widespread significance of microbial alterations in systemic inflammatory conditions. The blue cluster reflected mechanistic and interventional research on the AS-gut microbiota axis, featuring 24 keywords, including ankylosing spondylitis, microbiota, and HLA-B27, illustrating intricate interactions among genetic factors the microbiome and immune processes. The yellow cluster represented recent trends in causal inference and epidemiological studies, comprising 15 keyword, such as gut microbiota, Mendelian randomization, and autoimmune disease suggesting a transition from correlation-based analyses to causal investigation. Although these clusters were thematically distinct, they were interconnected across immune, microbial, and disease-phenotype dimensions, jointly forming the overarching research framework of this field.

The temporal analysis of keywords (Figure 6B) further illustrated the evolution of research hotspots. Early research primarily focused on immunological mechanisms in AS and related immune-mediated arthritis, with particular emphasis on HLA-B27 and inflammatory factors. In the intermediate phase, research progressively shifted toward links between gut dysbiosis and immune-mediated diseases, while exploring the potential of probiotics and microbiota-based interventions. More recently, the research focus has clearly shifted toward causal validation, with Mendelian randomization emerging as a key approach to clarify how alterations in the gut microbiota influence AS pathogenesis and progression via immune pathways.

The keyword burst analysis (Figure 6C) showed a clear progression in the field, from descriptive association studies to mechanistic research and, more recently, to translational exploration. Over the past 5 years (2021–2025), burst keywords have clustered predominantly around Mendelian randomization, fecal microbiota transplantation, indicating that early disease characterization and targeted microbiota-based interventions are emerging as key directions in current and future translational research.

## 4 Discussion

### 4.1 Overall research landscape

Bibliometric analysis indicates that research on AS and the gut microbiota has expanded rapidly since 2014, with sustained growth in publications and citations, reflecting the field’s transition from an exploratory niche to a significant research focus. China ranks first in publication output, while the United States and European countries play central roles in highly cited research and collaboration networks. Research themes have likewise evolved, shifting from association-driven studies to integrated mechanistic and translational investigations. The gut-joint axis has progressed from a conceptual hypothesis to a testable research framework, offering new avenues for microbiota-based biomarker discovery, prediction of treatment response, and precision microbiota-targeted interventions.

### 4.2 Evolution of research paradigms in the AS-gut microbiota field

Overall, research on AS and the gut microbiota has shown a clear progression, moving from investigations of immunoinflammatory mechanisms to association-based descriptions of dysbiosis, then to integrated mechanistic and interventional studies of the AS-gut microbiota axis, and ultimately to genetic tool-based causal inference research. It is important to distinguish that the bibliometric analysis in this study reflects research trends and thematic evolution, whereas interpretations regarding biological mechanisms are derived from the existing literature. The identification of a shift from descriptive to mechanistic and causal research is based on multiple bibliometric indicators, including keyword co-occurrence patterns, temporal evolution of keywords, and citation burst analysis. These indicators collectively reflect changes in research focus over time. However, it should be noted that bibliometric analyses capture publication trends and research attention, which may not always directly correspond to actual conceptual or methodological advances in the field.

#### 4.2.1 Cross-disease immune mechanisms linking the gut and AS

AS involves the spine, sacroiliac joints, entheses, and peripheral joints, and is often associated with extra-articular manifestations, most commonly anterior uveitis and intestinal inflammation (Zhu et al., 2019). A growing body of evidence indicates marked overlap in immunoinflammatory pathways among AS, inflammatory bowel disease, psoriatic arthritis, and axSpA, suggesting shared inflammatory drivers (Bragazzi et al., 2024). These diseases are closely associated with dysregulation of both innate and adaptive immune systems, characterized by excessive production of pro-inflammatory cytokines and aberrant activation of T helper cells. Approximately 50% of patients with AS exhibit subclinical intestinal inflammation, and about 10% eventually progress to clinically overt IBD (Costello et al., 2015). This observation provides strong evidence for the proximity of AS and IBD within the disease spectrum and supports their similarities in clinical presentation, immune phenotypes, and gut microbiota. Multiple studies have shown that these diseases are closely associated with aberrant activation of the IL-23/IL-17 axis, accompanied by sustained enhancement of Th17-related immune responses in intestinal inflammation (Hecquet et al., 2021; Amatya et al., 2017). The gut is considered a key site for immune priming in this axis. Evidence suggests that gut-activated T cells migrate to joint tissues via adhesion molecules, including αEβ7 and α4β7, and chemokine gradients, leading to sustained amplification of inflammatory responses in the axial skeleton (Dai et al., 2021). Based on this evidence, AS has been incorporated into the spectrum of gut-driven immune diseases. This framework not only reinforces the immunological foundation of the gut-joint axis but also implies that gut mucosal immune dysregulation may constitute a shared starting point for multiple immune-mediated diseases, offering new insights into cross-disease therapeutic approaches.

#### 4.2.2 Shared gut microbiota dysbiosis and barrier dysfunction across immune-mediated diseases

Increasing evidence indicates that patients with AS and related immune-mediated diseases exhibit marked changes in gut microbiota composition that are closely correlated with inflammation, disease activity, and clinical phenotypes (Su et al., 2024; Bragazzi et al., 2024). Additional evidence has identified a shared dysbiosis signature between IBD and spondyloarthropathy, characterized by depletion of SCFA-producing taxa, including *Faecalibacterium prausnitzii*, Roseburia, *Eubacterium hallii*, and Coprococcus spp., and enrichment of Proteobacteria, Streptococcus, Haemophilus, and Escherichia/Shigella. This shared microbiota profile supports common mucosal immune activation pathways between AS and IBD and offers a microbiological explanation for the high prevalence of subclinical intestinal inflammation in AS. Decreased microbial diversity and aberrant enrichment of specific taxa are frequently associated with increased intestinal permeability and reduced tight junction expression, which, in turn, amplify inflammatory signaling (Kinashi and Hase, 2021; Wang et al., 2024). In AS, intestinal expression of ZO-1 and occludin is markedly decreased. At the same time, modulating the pro-inflammatory microbiota can restore their expression, suppress LPS-TLR4 signaling, and reduce TNF-α, IL-17A, IL-6, and IFN-γ levels (Ding et al., 2023).

Microbial antigen translocation provides key pathological evidence linking gut dysbiosis to immune activation and joint inflammation. In patients with SpA, microbial DNA from the gastrointestinal or genitourinary tract has been detected in synovial cells, with a prevalence significantly higher than that observed in rheumatoid arthritis (Elwell et al., 2016). Overall, cross-barrier translocation of microbial antigens tightly links gut dysbiosis to systemic inflammation and joint lesions, reinforcing the pathological relevance of the gut-joint axis in AS.

#### 4.2.3 Gut microbiota changes in AS and treatment response

HLA-B27 has long been recognized as the principal genetic risk factor for AS, although its exact pathogenic mechanisms remain incompletely elucidated. Multiple hypotheses have been proposed regarding HLA-B27’s role in AS, including the arthritogenic peptide, protein misfolding, and cell-surface HLA-B27 homodimer hypotheses (Sharip and Kunz, 2020). More recently, interest has focused on the interplay between HLA-B27 and the gut microbiota. Evidence indicates that HLA-B27 transgenic mice undergo spontaneous gut microbiota remodeling, accompanied by intestinal inflammation and arthritis-like phenotypes. Moreover, distinct gut microbiota profiles markedly influence the severity of inflammatory responses in HLA-B27 models (Gill et al., 2018). These observations indicate that HLA-B27-associated genetic backgrounds can shape distinct gut microbial environments, modulating immune activation and participating in AS development. Building on this framework, the molecular mimicry hypothesis provides an integrated mechanistic explanation for the interactions among HLA-B27, the gut microbiota, and autoimmune inflammation (Zhang et al., 2018). Evidence indicates that certain bacteria enriched in the gut of AS patients, especially *Klebsiella pneumoniae*, possess antigenic peptide sequences highly homologous to HLA-B27-presented self-peptides or cartilage-related host structures. Such bacteria can trigger cross-reactive T-cell responses, converting antimicrobial immunity into autoimmune attacks on joints, the spine, and enthesis tissues. This mechanism underscores HLA-B27 as an immune response amplifier within specific microbial environments, further supporting a tripartite model driven by genetic, microbial, and immune interactions in AS. The gut microbiota may additionally contribute to AS immunopathology by inducing endoplasmic reticulum stress and promoting HLA-B27 misfolding (Gill et al., 2018). Adverse gut microbes or their metabolites can trigger the unfolded protein response or autophagy pathways, facilitating HLA-B27 misfolding and amplifying IL-23/IL-17 axis activity (Antoniou et al., 2019; Naama et al., 2023). Conversely, beneficial SCFA-producing taxa may suppress this process by preserving intestinal barrier integrity (Di Mattia et al., 2025).

From a therapeutic perspective, drug-related studies provide further evidence for the gut-joint axis. Nonsteroidal anti-inflammatory drugs, despite their widespread use for symptom control, have been shown to decrease beneficial bacteria, increase potentially pathogenic taxa, and worsen intestinal barrier dysfunction (Rogers and Aronoff, 2016). By contrast, conventional synthetic disease-modifying antirheumatic drugs partially restore SCFA-related taxa and reduce Proteobacteria abundance (Yemula and Sheikh, 2024). Anti-TNF-*α* biologics markedly reduce inflammatory activity in AS and remodel the gut microbiota, restoring microbial diversity and key taxa toward healthy-control levels, while decreasing potentially arthritogenic peptides and repairing tight junctions, thereby improving intestinal barrier integrity (Ditto et al., 2021; Yuan et al., 2024). IL-17 inhibitors influence fatty acid metabolism, fungal communities, and specific anaerobic taxa to varying degrees (Manasson et al., 2020). Fecal microbiota transplantation (FMT) restores gut microbial ecology. It has shown apparent efficacy in refractory Clostridioides difficile infection and specific inflammatory bowel disease, and antibiotic pretreatment can further facilitate the recovery of key microbial taxa (Khoruts and Sadowsky, 2016). Nevertheless, inconsistent results from FMT studies in humans suggest that microbiota-based interventions may exhibit substantial interindividual variability (Mauro et al., 2025). In addition, FMT carries safety concerns, including transient effects, the need for repeated transplantation, and the risk of pathogen transmission. Infectious complications after transplantation have been reported (Hohmann et al., 2014).

#### 4.2.4 From association to causality in AS-microbiome research

In recent years, research in this field has increasingly shifted from conventional association analyses to a causal inference framework. As large-scale genome-wide association and gut microbiome datasets have accumulated, Mendelian randomization (MR) has been used to assess potential causal relationships among gut microbiota, inflammatory pathways, immune phenotypes, and AS. By leveraging genetic variants as instrumental variables, MR studies mitigate confounding and reverse-causation bias, thereby providing more robust evidence for a causal role of the gut microbiota in AS onset (Wei et al., 2024). Notably, despite heterogeneity in the specific taxa identified across studies, functional metagenomic evidence consistently shows enrichment of AS-associated microbiota in pro-inflammatory pathways, including LPS biosynthesis, flagellin, secretion systems, and energy metabolism, indicating a functionally convergent dysbiotic state. This functional convergence provides strong biological plausibility for the directional relationships suggested by MR analyses.

### 4.3 Emerging trends in the AS-gut microbiota field

The emerging themes presented in this section reflect recent shifts in research focus identified through keyword evolution and citation burst analysis. These patterns indicate areas of growing interest within the field and highlight directions that are likely to shape future research. Therefore, the following subsections summarize key emerging topics and evolving research priorities in the AS–gut microbiota field.

#### 4.3.1 Functional convergence of gut microbial pathways in AS

In AS, gut microbiota alterations extend beyond composition to functional pathways, including enhanced LPS biosynthesis, increased expression of flagellin and secretion system proteins, and heightened activity in energy metabolism pathways, indicating a shift in the gut ecosystem toward a pro-inflammatory state (Yemula and Sheikh, 2024). Notably, despite heterogeneity in the specific taxa identified across studies, functional annotation consistently shows enrichment for pro-inflammatory functions, suggesting that AS-associated gut microbiota may exhibit functional convergence that synergistically contributes to immune activation (Mauro et al., 2021).

SCFAs derived from the gut microbiota enhance tight junction expression and reduce zonulin levels through G protein-coupled receptor signaling and HDAC inhibition, thereby preserving barrier integrity and limiting bacterial product translocation. In addition, SCFAs modulate the Th17/Treg balance, suppress the IL-23/IL-17 axis, inhibit NLRP3 inflammasome activation, and influence osteoclast metabolism, thereby coordinating inflammatory responses and bone remodeling and underscoring their immunometabolic regulatory potential in AS (Asquith et al., 2017; Lucas et al., 2018).

Tryptophan (Trp) is metabolized by the gut microbiota into indole and its derivatives (IPA, IAA, and IAld), which modulate intestinal barrier integrity and immune homeostasis by activating AhR or PXR. IPA strengthens tight junctions and inhibits TNF-α, whereas IAA and IAld stimulate IL-22 production by ILCs, thereby promoting mucosal repair (Gutierrez-Vazquez and Quintana, 2018; Korecka et al., 2016; Lanis et al., 2017). Metagenomic and metabolomic evidence indicate that SpA patients have decreased Trp biosynthetic capacity but increased metabolic activity. Dysregulation of the Trp-indole pathway is strongly linked to disease progression, highlighting its potential as a pathogenic pathway and intervention target (Yin et al., 2020). TMAO may also promote ZO-1 downregulation and tight junction disruption via NLRP3 activation, thereby increasing intestinal permeability and amplifying inflammatory signaling loops (Scalise et al., 2021). Collectively, these metabolic pathways link the gut microbiota to immune activation, barrier impairment, and systemic inflammation in AS, suggesting their potential as diagnostic biomarkers and therapeutic targets.

#### 4.3.2 Zonulin-mediated intestinal barrier dysfunction in AS

Increasing evidence indicates that zonulin-mediated intestinal barrier dysfunction is a key molecular basis for gut-joint axis dysregulation in AS. Even in AS patients without overt gastrointestinal symptoms and with essentially normal endoscopic morphology, elevated zonulin levels in serum or intestinal mucosa are detectable, along with increased LPS and LPS-binding protein levels, suggesting subclinical intestinal barrier impairment (Ciccia et al., 2017). As a critical regulator of tight junctions, increased zonulin expression disrupts ZO-1 and occludin structure and function, thereby increasing permeability of intestinal epithelial and vascular barriers. Zonulin-driven barrier disruption promotes translocation of microbe-associated molecular patterns across the intestinal barrier, activating TLR-4, NOD-like receptors, and NLRP3 inflammasome signaling, which, in turn, amplifies IL-23/IL-17 axis activity and TNF-α release, driving mucosal and systemic immune activation (Stevens et al., 2018). Notably, longitudinal studies in SpA-related animal models indicate that zonulin elevation occurs primarily during the preclinical or early disease phase and partially declines to baseline during acute arthritis, implying that intestinal permeability changes are more critical for disease initiation than for maintenance (Hecquet et al., 2023). Moreover, anti-inflammatory or biologic treatments partially reduce zonulin expression, restore tight junction integrity, and improve gut microbiota composition and inflammatory factors, providing interventional evidence for the functional role of zonulin in the AS gut-joint axis (Tajik et al., 2020). Overall, as a pivotal molecular node linking gut dysbiosis, barrier dysfunction, and immune activation, zonulin enhances understanding of AS pathogenesis and highlights its potential as a disease activity biomarker and therapeutic target.

#### 4.3.3 TNF Signaling at the Interface of gut microbiota and joint inflammation

Recent evidence has repositioned TNF signaling within the gut microbiota-barrier-immune axis, indicating that it functions both as an amplifier of inflammation and as a critical mediator through which microbial dysbiosis drives immune abnormalities. Evidence suggests that anti-TNF therapy not only markedly improves clinical manifestations of AS but also partially normalizes gut microbiota composition. Metagenomic evidence shows that TNF inhibitor therapy reduces potentially arthritogenic bacterial peptides in the gut of SpA patients and shifts microbial composition toward healthy-control profiles, suggesting that anti-TNF therapy may, at least in part, exert anti-inflammatory effects by modulating the gut microbiota (Ditto et al., 2021).

Nevertheless, a considerable proportion of patients fail to respond to anti-TNF therapy. Evidence indicates that baseline gut microbiota features are closely linked to TNF inhibitor efficacy, and specific microbial configurations may serve as predictive biomarkers of therapeutic response (Bazin et al., 2018). Moreover, compared with IL-17 inhibitors, TNF inhibitors have a milder, more stable effect on the gut microbiota, which may better preserve intestinal immune homeostasis and potentially reduce the risk of gut-related adverse events (Manasson et al., 2020).

### 4.4 Limitations

Bibliometric methods rely on published metadata and cannot account for differences in study design, sequencing platforms, or clinical heterogeneity. Therefore, the results reflect research trends and intellectual development rather than direct biological or clinical conclusions. Although the final search was conducted in February 2026, data from 2026 were excluded from the annual trend analysis due to incomplete coverage. While this ensures comparability across years, it may limit the ability to reflect the most recent developments. Future updates with complete annual data will be necessary to capture ongoing trends more accurately. Second, only English-language publications were included, which may introduce language bias and limit the global representativeness of the findings. Further research is needed to address this limitation.

## 5 Conclusion

In summary, this bibliometric analysis indicates that research on AS and the gut microbiota has shifted from descriptive associations to mechanistic and causal investigation. Based on the existing literature, accumulating evidence highlights the potential involvement of the gut–joint axis in AS, including microbial dysbiosis, impaired intestinal barrier function, and mucosal immune responses. Functional and multi-omics studies increasingly highlight pathway-level analyses of microbial alterations rather than single-taxon effects. Mendelian randomization approaches have been increasingly applied to explore potential causal relationships. Based on the bibliometric findings, emerging trends identified through keyword evolution and citation burst analyses suggest growing interest in causal inference and microbiota-based interventions. Future studies should further advance these emerging directions, with greater emphasis on functional and pathway-level analyses to enhance the translational relevance of microbiota research in AS.

## Data Availability

The raw data supporting the conclusions of this article will be made available by the authors, without undue reservation.

## References

[ref1] Amatya N. Garg A. V. Gaffen S. L. (2017). IL-17 Signaling: the Yin and the Yang. Trends Immunol. 38, 310–322. doi: 10.1016/j.it.2017.01.006 28254169 PMC5411326

[ref2] Antoniou A. N. Lenart I. Kriston-Vizi J. Iwawaki T. Turmaine M. McHugh K. . (2019). Salmonella exploits HLA-B27 and host unfolded protein responses to promote intracellular replication. Ann. Rheum. Dis. 78, 74–82. doi: 10.1136/annrheumdis-2018-213532, 30355574 PMC6317449

[ref3] Asquith M. Davin S. Stauffer P. Michell C. Janowitz C. Lin P. . (2017). Intestinal metabolites are profoundly altered in the context of HLA-B27 expression and functionally modulate disease in a rat model of Spondyloarthritis. Arthritis Rheumatol. 69, 1984–1995. doi: 10.1002/art.40183, 28622455 PMC5623151

[ref4] Bazin T. Hooks K. B. Barnetche T. Truchetet M. E. Enaud R. Richez C. . (2018). Microbiota composition may predict anti-Tnf alpha response in Spondyloarthritis patients: an exploratory study. Sci. Rep. 8:5446. doi: 10.1038/s41598-018-23571-4, 29615661 PMC5882885

[ref5] Berland M. Meslier V. Berreira Ibraim S. Le Chatelier E. Pons N. Maziers N. . (2023). Both disease activity and HLA-B27 status are associated with gut microbiome dysbiosis in Spondyloarthritis patients. Arthritis Rheumatol. 75, 41–52. doi: 10.1002/art.42289, 35818337 PMC10099252

[ref6] Bragazzi M. C. Pianigiani F. Venere R. Ridola L. (2024). Dysbiosis in inflammatory bowel disease and Spondyloarthritis: still a long way to go? J. Clin. Med. 13:2237. doi: 10.3390/jcm13082237, 38673510 PMC11050776

[ref7] Breban M. Tap J. Leboime A. Said-Nahal R. Langella P. Chiocchia G. . (2017). Faecal microbiota study reveals specific dysbiosis in spondyloarthritis. Ann. Rheum. Dis. 76, 1614–1622. doi: 10.1136/annrheumdis-2016-211064 28606969

[ref8] Ciccia F. Guggino G. Rizzo A. Alessandro R. Luchetti M. M. Milling S. . (2017). Dysbiosis and zonulin upregulation alter gut epithelial and vascular barriers in patients with ankylosing spondylitis. Ann. Rheum. Dis. 76, 1123–1132. doi: 10.1136/annrheumdis-2016-210000 28069576 PMC6599509

[ref9] Costello M. E. Ciccia F. Willner D. Warrington N. Robinson P. C. Gardiner B. . (2015). Brief Report: Intestinal Dysbiosis in Ankylosing Spondylitis. Arthritis Rheumatol. 67, 686–691., 25417597 10.1002/art.38967

[ref10] Dai B. Hackney J. A. Ichikawa R. Nguyen A. Elstrott J. Orozco L. D. . (2021). Dual targeting of lymphocyte homing and retention through alpha4beta7 and alphaEbeta7 inhibition in inflammatory bowel disease. Cell Rep Med. 2:100381. doi: 10.1016/j.xcrm.2021.100381, 34467254 PMC8385326

[ref11] Di Mattia M. Sallese M. Lopetuso L. R. (2025). The interplay between gut microbiota and the unfolded protein response: implications for intestinal homeostasis preservation and dysbiosis-related diseases. Microb. Pathog. 200:107279. doi: 10.1016/j.micpath.2025.107279 39761770

[ref12] Ding M. H. Xu P. G. Wang Y. Ren B. D. Zhang J. L. (2023). Resveratrol attenuates ankylosing spondylitis in mice by inhibiting the TLR4/NF-kappaB/NLRP3 pathway and regulating gut microbiota. Immunol. Investig. 52, 194–209. doi: 10.1080/08820139.2022.2154162, 36548483

[ref13] Ditto M. C. Parisi S. Landolfi G. Borrelli R. Realmuto C. Finucci A. . (2021). Intestinal microbiota changes induced by TNF-inhibitors in IBD-related spondyloarthritis. RMD Open 7:e001755. doi: 10.1136/rmdopen-2021-001755, 34489323 PMC8422478

[ref14] Elwell C. Mirrashidi K. Engel J. (2016). Chlamydia cell biology and pathogenesis. Nat. Rev. Microbiol. 14, 385–400. doi: 10.1038/nrmicro.2016.30, 27108705 PMC4886739

[ref15] Gill T. Asquith M. Brooks S. R. Rosenbaum J. T. Colbert R. A. (2018). Effects of HLA-B27 on gut microbiota in experimental Spondyloarthritis implicate an ecological model of dysbiosis. Arthritis Rheumatol. 70, 555–565. doi: 10.1002/art.40405, 29287307 PMC6101666

[ref16] Gracey E. Vereecke L. McGovern D. Frohling M. Schett G. Danese S. . (2020). Revisiting the gut-joint axis: links between gut inflammation and spondyloarthritis. Nat. Rev. Rheumatol. 16, 415–433. doi: 10.1038/s41584-020-0454-9, 32661321

[ref17] Gutierrez-Vazquez C. Quintana F. J. (2018). Regulation of the immune response by the aryl hydrocarbon receptor. Immunity 48, 19–33. doi: 10.1016/j.immuni.2017.12.012, 29343438 PMC5777317

[ref18] Hecquet S. Totoson P. Martin H. Algros M. P. Saas P. Pais-de-Barros J. P. . (2023). Increased gut permeability and intestinal inflammation precede arthritis onset in the adjuvant-induced model of arthritis. Arthritis Res. Ther. 25:95. doi: 10.1186/s13075-023-03069-9, 37280714 PMC10242991

[ref19] Hecquet S. Totoson P. Martin H. Prati C. Wendling D. Demougeot C. . (2021). Intestinal permeability in spondyloarthritis and rheumatoid arthritis: a systematic review of the literature. Semin. Arthritis Rheum. 51, 712–718. doi: 10.1016/j.semarthrit.2021.04.015, 34139524

[ref20] Hohmann E. L. Ananthakrishnan A. N. Deshpande V. (2014). Case Records of the Massachusetts General Hospital. Case 25-2014. A 37-year-old man with ulcerative colitis and bloody diarrhea. N. Engl. J. Med. 371, 668–675. doi: 10.1056/NEJMcpc1400842, 25119613

[ref21] Khoruts A. Sadowsky M. J. (2016). Understanding the mechanisms of faecal microbiota transplantation. Nat. Rev. Gastroenterol. Hepatol. 13, 508–516. doi: 10.1038/nrgastro.2016.98 27329806 PMC5909819

[ref22] Kinashi Y. Hase K. (2021). Partners in Leaky gut Syndrome: intestinal dysbiosis and autoimmunity. Front. Immunol. 12:673708. doi: 10.3389/fimmu.2021.673708, 33968085 PMC8100306

[ref9002] Klingberg E. Magnusson M. K. Strid H. Deminger A. Ståhl A. Sundin J. . (2019). A distinct gut microbiota composition in patients with ankylosing spondylitis is associated with increased levels of fecal calprotectin. Arthritis Res Ther. 21:248., 31771630 10.1186/s13075-019-2018-4 PMC6880506

[ref23] Korecka A. Dona A. Lahiri S. Tett A. J. Al-Asmakh M. Braniste V. . (2016). Bidirectional communication between the aryl hydrocarbon receptor (AhR) and the microbiome tunes host metabolism. NPJ Biofilms Microbiomes 2:16014. doi: 10.1038/npjbiofilms.2016.14, 28721249 PMC5515264

[ref24] Lanis J. M. Alexeev E. E. Curtis V. F. Kitzenberg D. A. Kao D. J. Battista K. D. . (2017). Tryptophan metabolite activation of the aryl hydrocarbon receptor regulates IL-10 receptor expression on intestinal epithelia. Mucosal Immunol. 10, 1133–1144. doi: 10.1038/mi.2016.133, 28098246 PMC5515702

[ref9001] Lin P. Bach M. Asquith M. Lee A. Y. Akileswaran L. Stauffer P. . (2014). HLA-B27 and human β2-microglobulin affect the gut microbiota of transgenic rats. PLoS One. 9:e105684., 25140823 10.1371/journal.pone.0105684 PMC4139385

[ref25] Lobiuc A. Groppa L. Chislari L. Russu E. Homitchi M. Ciorescu C. . (2025). Gut microbiota and ankylosing spondylitis: current insights and future challenges. Microb Cell. 12, 210–230. doi: 10.15698/mic2025.08.857, 40904690 PMC12404692

[ref26] Lucas S. Omata Y. Hofmann J. Bottcher M. Iljazovic A. Sarter K. . (2018). Short-chain fatty acids regulate systemic bone mass and protect from pathological bone loss. Nat. Commun. 9:55. doi: 10.1038/s41467-017-02490-4, 29302038 PMC5754356

[ref27] Manasson J. Wallach D. S. Guggino G. Stapylton M. Badri M. H. Solomon G. . (2020). Interleukin-17 inhibition in Spondyloarthritis is associated with subclinical gut microbiome perturbations and a distinctive Interleukin-25-driven intestinal inflammation. Arthritis Rheumatol. 72, 645–657. doi: 10.1002/art.41169, 31729183 PMC7113119

[ref28] Mauro D. Cai B. Ciancio A. Forte G. Gandolfo S. Thomas R. . (2025). The role of the gut and intestinal dysbiosis in the pathogenesis of spondyloarthritis. Joint Bone Spine 92:105923. doi: 10.1016/j.jbspin.2025.105923, 40404007

[ref29] Mauro D. Thomas R. Guggino G. Lories R. Brown M. A. Ciccia F. (2021). Ankylosing spondylitis: an autoimmune or autoinflammatory disease? Nat. Rev. Rheumatol. 17, 387–404. doi: 10.1038/s41584-021-00625-y 34113018

[ref30] Min H. K. Na H. S. Jhun J. Lee S. Y. Choi S. S. Park G. E. . (2023). Identification of gut dysbiosis in axial spondyloarthritis patients and improvement of experimental ankylosing spondyloarthritis by microbiome-derived butyrate with immune-modulating function. Front. Immunol. 14:1096565. doi: 10.3389/fimmu.2023.1096565, 37143677 PMC10152063

[ref31] Naama M. Telpaz S. Awad A. Ben-Simon S. Harshuk-Shabso S. Modilevsky S. . (2023). Autophagy controls mucus secretion from intestinal goblet cells by alleviating ER stress. Cell Host Microbe 31, 433–446.e4. doi: 10.1016/j.chom.2023.01.006, 36738733 PMC10016318

[ref32] Ng B. C. K. Lassere M. (2025). The role of the gastrointestinal microbiome on rheumatoid arthritis, psoriatic arthritis, ankylosing spondylitis and reactive arthritis: a systematic review. Semin. Arthritis Rheum. 70:152574. doi: 10.1016/j.semarthrit.2024.152574, 39644691

[ref33] Qaiyum Z. Lim M. Inman R. D. (2021). The gut-joint axis in spondyloarthritis: immunological, microbial, and clinical insights. Semin. Immunopathol. 43, 173–192. doi: 10.1007/s00281-021-00845-0, 33625549

[ref34] Rogers M. A. M. Aronoff D. M. (2016). The influence of non-steroidal anti-inflammatory drugs on the gut microbiome. Clin. Microbiol. Infect. 22, 178.e1–178.e9. doi: 10.1016/j.cmi.2015.10.003 PMC4754147 26482265

[ref35] Scalise G. Ciancio A. Mauro D. Ciccia F. (2021). Intestinal microbial metabolites in ankylosing spondylitis. J. Clin. Med. 10:3354. doi: 10.3390/jcm10153354, 34362137 PMC8347740

[ref36] Sharip A. Kunz J. (2020). Understanding the pathogenesis of Spondyloarthritis. Biomolecules. 10:1461. doi: 10.3390/biom10101461 33092023 PMC7588965

[ref37] Sieper J. Poddubnyy D. (2017). Axial spondyloarthritis. Lancet 390, 73–84. doi: 10.1016/S0140-6736(16)31591-4 28110981

[ref38] Stevens B. R. Goel R. Seungbum K. Richards E. M. Holbert R. C. Pepine C. J. . (2018). Increased human intestinal barrier permeability plasma biomarkers zonulin and FABP2 correlated with plasma LPS and altered gut microbiome in anxiety or depression. Gut 67, 1555.2–1555.7. doi: 10.1136/gutjnl-2017-314759 PMC5851874 28814485

[ref39] Su Q. Y. Zhang Y. Qiao D. Song X. Shi Y. Wang Z. . (2024). Gut microbiota dysbiosis in ankylosing spondylitis: a systematic review and meta-analysis. Front. Cell. Infect. Microbiol. 14:1376525. doi: 10.3389/fcimb.2024.1376525, 39421642 PMC11484232

[ref40] Tajik N. Frech M. Schulz O. Schalter F. Lucas S. Azizov V. . (2020). Targeting zonulin and intestinal epithelial barrier function to prevent onset of arthritis. Nat. Commun. 11:1995. doi: 10.1038/s41467-020-15831-7, 32332732 PMC7181728

[ref41] Wang H. L. Cai Y. S. Wu W. Q. Zhang M. M. Dai Y. Wang Q. W. (2024). Exploring the role of gut microbiome in autoimmune diseases: a comprehensive review. Autoimmun. Rev. 23:103654. doi: 10.1016/j.autrev.2024.103654 39384149

[ref42] Wei Y. Zhang S. Shao F. Sun Y. (2025). Ankylosing spondylitis: from pathogenesis to therapy. Int. Immunopharmacol. 145:113709. doi: 10.1016/j.intimp.2024.113709 39644789

[ref43] Wei J. N. Zhu X. Y. Wang J. X. Yang K. Z. Chen J. Y. Li J. L. . (2024). Mendelian randomization studies in ankylosing spondylitis: a systematic review. Int. J. Rheum. Dis. 27:e15408. doi: 10.1111/1756-185X.15408 39503365

[ref9004] Wu H. J. Ivanov I. I. Darce J. Hattori K. Shima T. Umesaki Y. . (2010). Gut-residing segmented filamentous bacteria drive autoimmune arthritis via T helper 17 cells. Immunity. 32, 815–27., 20620945 10.1016/j.immuni.2010.06.001 PMC2904693

[ref44] Yemula N. Sheikh R. (2024). Gut microbiota in axial spondyloarthritis: genetics, medications and future treatments. ARP Rheumatol. 3, 216–225. doi: 10.63032/WUII1201 39243363

[ref45] Yin J. Sternes P. R. Wang M. Song J. Morrison M. Li T. . (2020). Shotgun metagenomics reveals an enrichment of potentially cross-reactive bacterial epitopes in ankylosing spondylitis patients, as well as the effects of TNFi therapy upon microbiome composition. Ann. Rheum. Dis. 79, 132–140. doi: 10.1136/annrheumdis-2019-215763, 31662318

[ref46] Yuan Y. X. Feng S. R. Wu A. Y. Wu W. H. Tian P. Chen A. Z. . (2024). Influence of TNF-α inhibitors on gut microbiota and immune modulation in treating ankylosing spondylitis: insights into therapeutic mechanisms and clinical implications. J. Inflamm. Res. 17, 11741–11752. doi: 10.2147/JIR.S496991, 39741755 PMC11687288

[ref47] Zhang Y. Liu W. Lai J. Zeng H. (2024). Genetic associations in ankylosing spondylitis: circulating proteins as drug targets and biomarkers. Front. Immunol. 15:1394438. doi: 10.3389/fimmu.2024.1394438, 38835753 PMC11148386

[ref48] Zhang L. Zhang Y. J. Chen J. Huang X. L. Fang G. S. Yang L. J. . (2018). The association of HLA-B27 and *Klebsiella pneumoniae* in ankylosing spondylitis: a systematic review. Microb. Pathog. 117, 49–54. doi: 10.1016/j.micpath.2018.02.020, 29438717

[ref9003] Zhou C. Zhao H. Xiao X. Y. Chen B. D. Guo R. J. Wang Q. . (2016). Metagenomic profiling of the pro-inflammatory gut microbiota in ankylosing spondylitis. J Autoimmun. 107:102360., 31806420 10.1016/j.jaut.2019.102360

[ref49] Zhu W. He X. Cheng K. Zhang L. Chen D. Wang X. . (2019). Ankylosing spondylitis: etiology, pathogenesis, and treatments. Bone Res. 7:22. doi: 10.1038/s41413-019-0057-8, 31666997 PMC6804882

