# Supplementary material for: Gut microbiota and ankylosing spondylitis: mechanisms, functional pathways, and research trends
Source: Front Microbiol. 2026 May 13;17:1828220. doi: 10.3389/fmicb.2026.1828220 (PMC13212314; doi:10.3389/fmicb.2026.1828220)
Supplement: Supplementary file 1 [file Table_1.DOCX]

**Search query**

Web of Science Core Collection (WOSCC):

TS=("gut microbiota" OR "intestinal microbiota" OR "fecal microbiota" OR "gastrointestinal microbiota" OR "gut microbiome" OR "intestinal microbiome" OR "fecal microbiome" OR "gastrointestinal microbiome" OR "intestinal bacteria" OR "gut bacteria" OR "fecal bacteria" OR "gastrointestinal bacteria" OR "intestinal flora" OR "gut flora" OR "fecal flora" OR "gastrointestinal flora" OR "gut microflora" OR "intestinal microflora" OR "fecal microflora" OR "gastrointestinal microflora") AND TS=("Ankylosing spondylitis" OR "axial spondyloarthritis" OR "axSpA" OR "spondyloarthritis" OR "SpA")

SCOPUS:

TITLE-ABS-KEY("gut microbiota" OR "intestinal microbiota" OR "fecal microbiota" OR "gastrointestinal microbiota" OR "gut microbiome" OR "intestinal microbiome" OR "fecal microbiome" OR "gastrointestinal microbiome" OR "intestinal bacteria" OR "gut bacteria" OR "fecal bacteria" OR "gastrointestinal bacteria" OR "intestinal flora" OR "gut flora" OR "fecal flora" OR "gastrointestinal flora" OR "gut microflora" OR "intestinal microflora" OR "fecal microflora" OR "gastrointestinal microflora") AND TITLE-ABS-KEY("Ankylosing spondylitis" OR "axial spondyloarthritis" OR "axSpA" OR "spondyloarthritis" OR "SpA")

PUBMED:

("gut microbiota"[Title/Abstract] OR "intestinal microbiota"[Title/Abstract] OR "fecal microbiota"[Title/Abstract] OR "gastrointestinal microbiota"[Title/Abstract] OR "gut microbiome"[Title/Abstract] OR "intestinal microbiome"[Title/Abstract] OR "fecal microbiome"[Title/Abstract] OR "gastrointestinal microbiome"[Title/Abstract] OR "intestinal bacteria"[Title/Abstract] OR "gut bacteria"[Title/Abstract] OR "fecal bacteria"[Title/Abstract] OR "gastrointestinal bacteria"[Title/Abstract] OR "intestinal flora"[Title/Abstract] OR "gut flora"[Title/Abstract] OR "fecal flora"[Title/Abstract] OR "gastrointestinal flora"[Title/Abstract] OR "gut microflora"[Title/Abstract] OR "intestinal microflora"[Title/Abstract] OR "fecal microflora"[Title/Abstract] OR "gastrointestinal microflora"[Title/Abstract]) AND ("Ankylosing spondylitis"[Title/Abstract] OR "axial spondyloarthritis"[Title/Abstract] OR "axSpA"[Title/Abstract] OR "spondyloarthritis"[Title/Abstract] OR "SpA"[Title/Abstract])

**Inclusion and exclusion criteria**

To minimize potential bias due to database updates, all literature searches were conducted on February 18, 2026. Two reviewers independently screened the retrieved records, with an inter-reviewer agreement rate of 96%. Discrepancies were resolved through discussion with a third reviewer until consensus was reached.

The exclusion criteria were as follows:

(1) Publication type: letters, editorials, errata, conference abstracts, case reports, and retracted articles were excluded;

(2) Duplicate records: duplicate publications were identified and removed based on DOI, title, and author–year matching;

(3) Language restriction: only English-language publications were included;

(4) Relevance: studies not directly related to ankylosing spondylitis and gut microbiota, or only marginally mentioning relevant terms, were excluded;

(5) Data completeness: records lacking essential bibliographic information (e.g., authors, affiliations, publication year, or citation data) were excluded.
